# Author Correction: Quasiparticle Andreev scattering in the *ν* = 1/3 fractional quantum Hall regime

**DOI:** 10.1038/s41467-024-45492-9

**Published:** 2024-02-05

**Authors:** P. Glidic, O. Maillet, C. Piquard, A. Aassime, A. Cavanna, Y. Jin, U. Gennser, A. Anthore, F. Pierre

**Affiliations:** 1 Université Paris-Saclay, CNRS, Centre de Nanosciences et de Nanotechnologies, 91120 Palaiseau, France; 2 Université Paris Cité, CNRS, Centre de Nanosciences et de Nanotechnologies, F-91120 Palaiseau, France

**Keywords:** Quantum Hall, Two-dimensional materials

Correction to: *Nature Communications* 10.1038/s41467-023-36080-4, published online 31 January 2023

The original version of this Article contained an error in the computation of the dc tunnelling current across the central (analyser) quantum point contact, which did not affect the conclusions but led to incorrect data being presented in Figures 3(c,d), 4, 9(d,e,f) and 10(c,d). The source of the error is described in the following.

When analysing the raw data, the dc tunnelling current *I*_T_, calculated correctly at negative bias, was by mistake duplicated with an opposite sign at positive bias. In most cases, the changes at *I*_T_>0 are hardly discernible since a canonical quantum point contact is symmetric. However, it is visible for the device tuning initially shown in the original Figure 3(c) and, consequently, for the original Figure 4. Furthermore, this resulted in an error in the displayed dc transmission ratio *τ*_A_, shown in the insets of the original Figure 3(c) and Figure 10(d).

Because a bias voltage asymmetry in the tunnelling current is not standard nor representative, the authors have chosen to display in the new Figure 3(c) the second data set obtained concomitantly using the other quasiparticle source (the top-left quantum point contact) with the same overall gate voltage tuning of the device. The data using the bottom-right quantum point contact as a source (displayed in the original version of Figure 3(c)) is now shown in the new Figure 9(b) with the corrected *I*_T_ and its associated dc transmission ratio *τ*_A_.

The correct version of Figure 3(a, c) shows previously unshown data using the top-left quantum point contact as a source. The correct version of Figure 3(d), corresponding to the same measurements as in the original version of the Article, now shows the corrected data analysis at *I*_T_>0 for both the main panel and inset. The correct version of Figure 3 is:
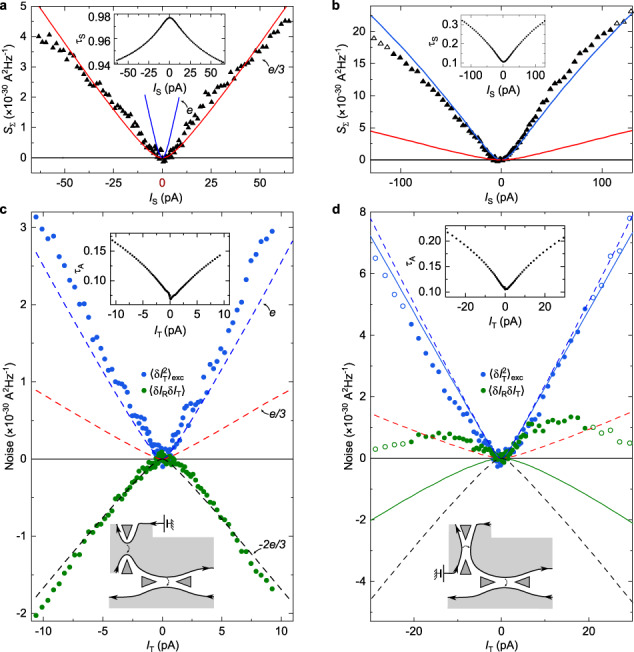


which replaces the previous incorrect version:
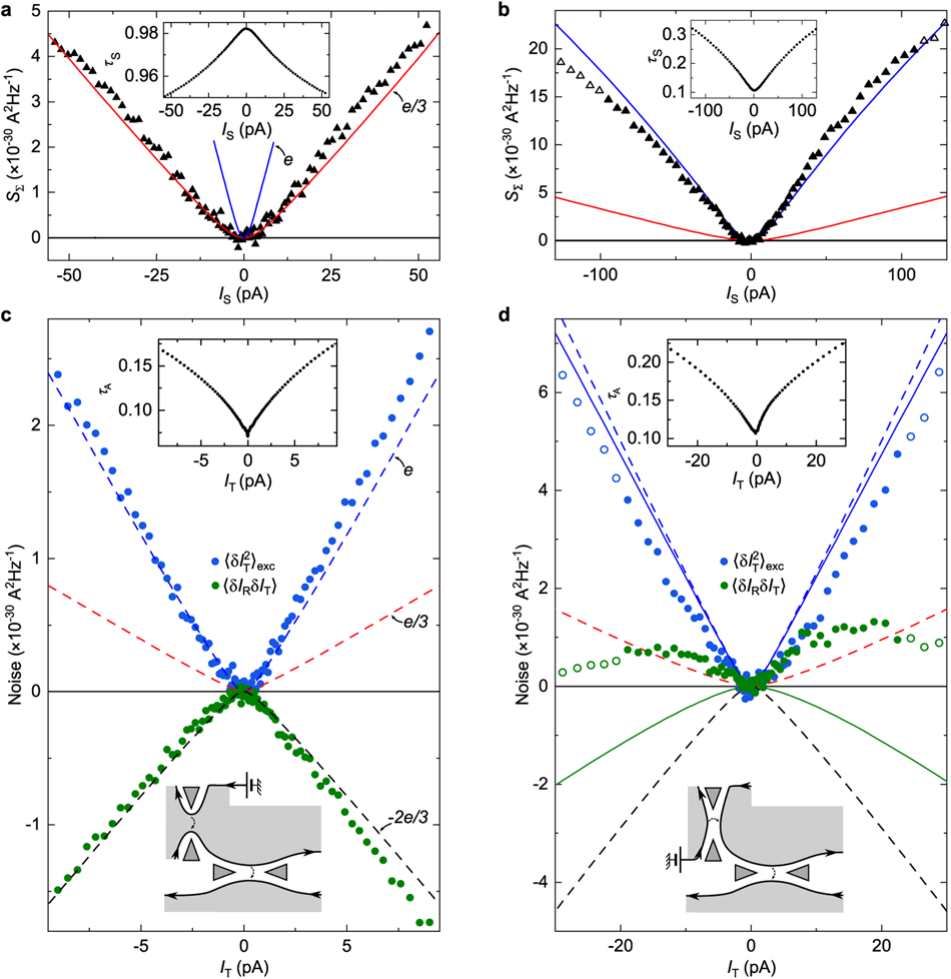


The correct version of Figure 4, corresponding to the same measurements as in the original version of the Article but with the corrected data analysis at *I*_T_>0, is:
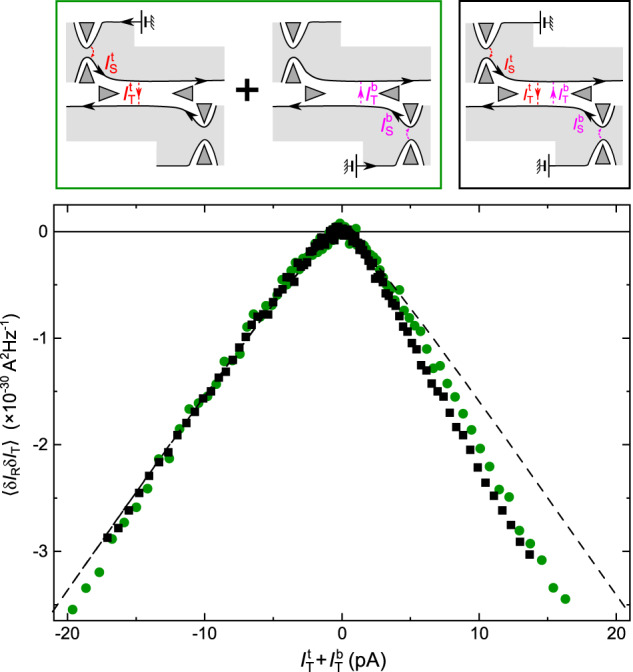


which replaces the previous incorrect version:
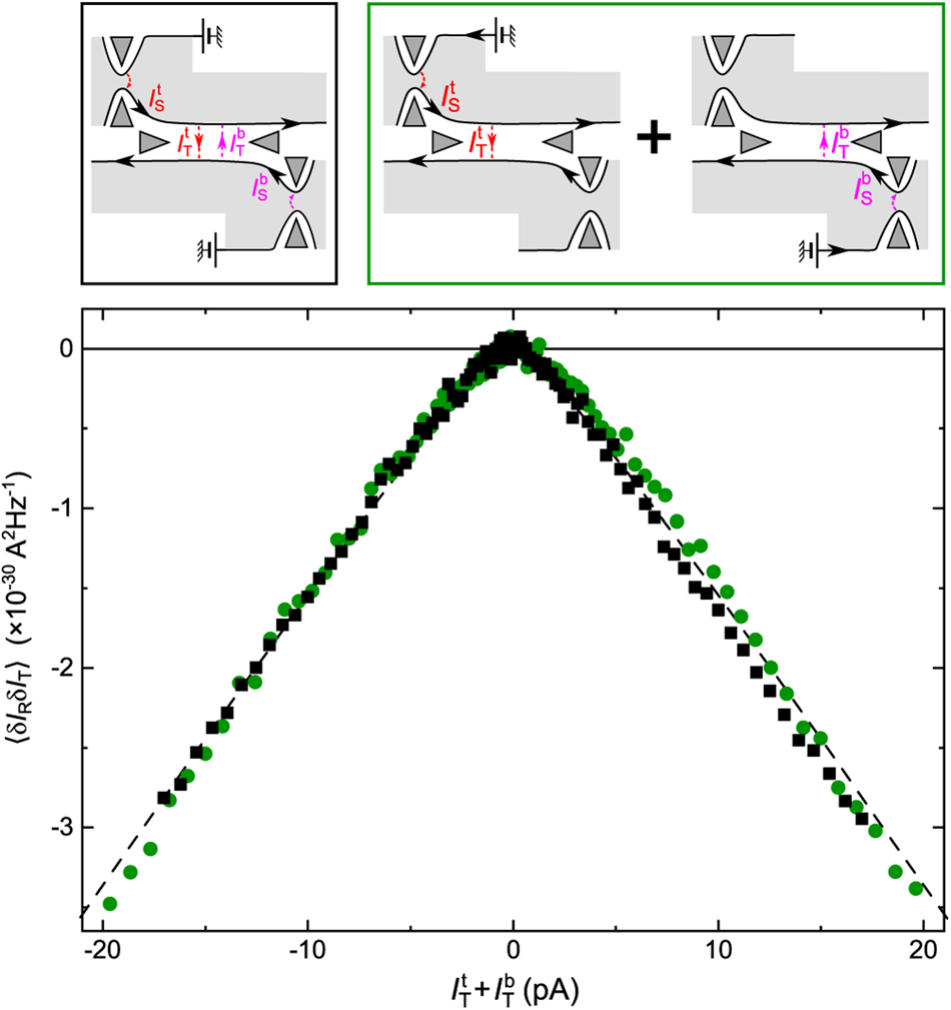


The correct version of Figure 9(e), corresponding to the measurements previously displayed in Figure 3(c) in the original version of the Article, now shows the corrected data analysis at *I*_T_>0 with the corrected *τ*_A_ in the inset. The correct version of Figure 9(d, f), corresponding to the same measurements as in the original version of the Article, now shows the corrected data analysis at *I*_T_>0. The correct version of Figure 9 is:
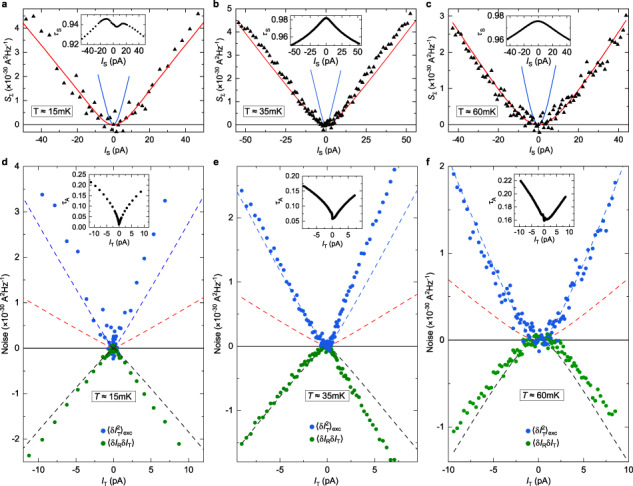


which replaces the previous incorrect version:
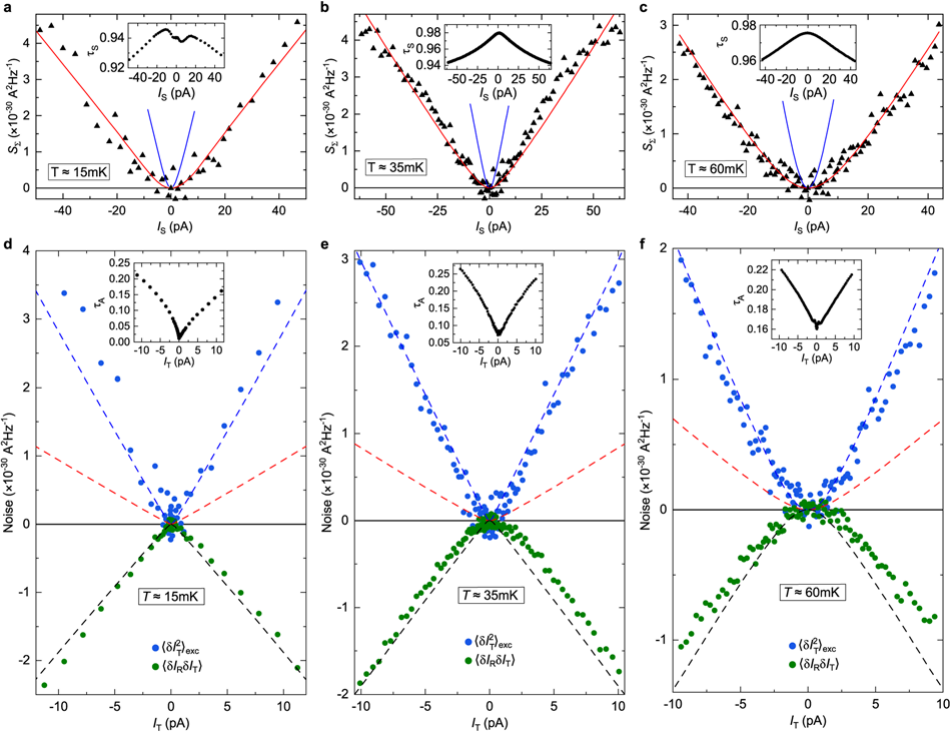


The correct version of Figure 10(c, d), corresponding to the same measurements as in the original version of the Article, now shows the corrected data analysis at *I*_T_>0 with the corrected *τ*_A_ in the inset of panel (d). The correct version of Figure 10 is:
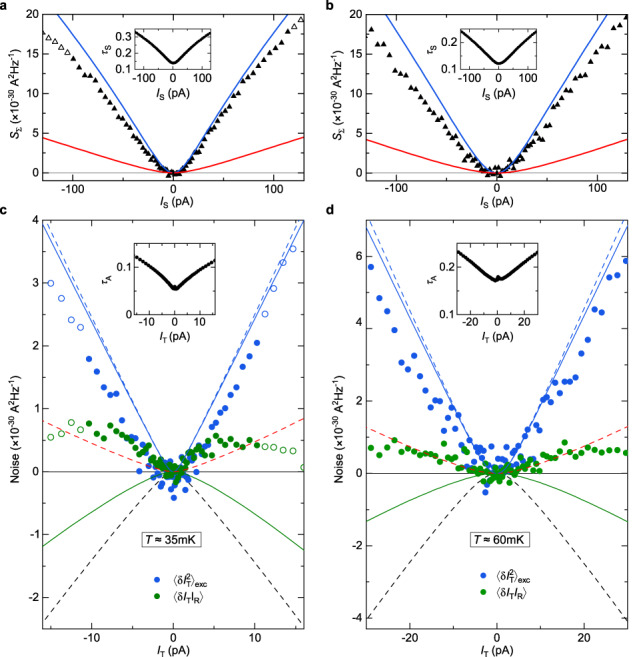


which replaces the previous incorrect version:
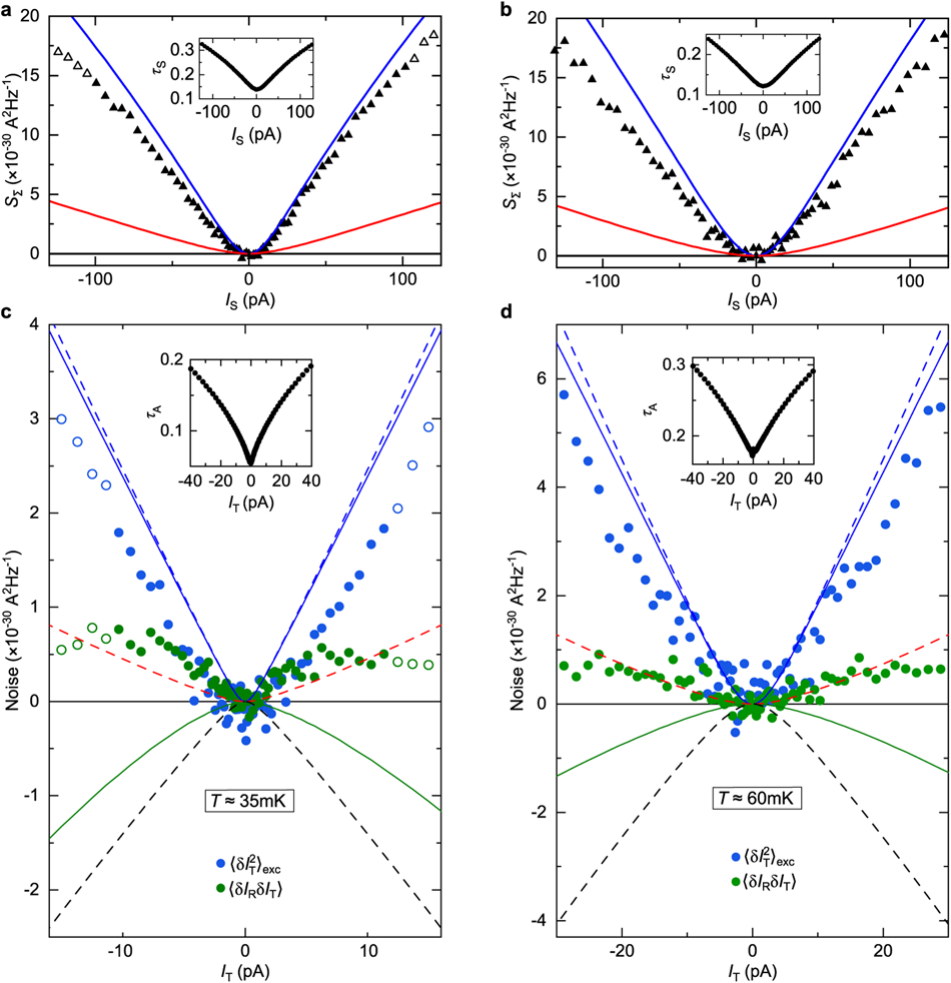


To reflect the changes in the figures, the following modifications to the text have been made.

The sentence “see Supplementary Information for a comparison with another gate voltage tuning of the device that exhibits a more canonical behavior at positive tunnel current.” has been added at the end of the caption of Figure 4.

In the caption of Figure 9, the first sentence “The source QPC is here located on the opposite side of the analyzer than for the data shown in Fig. 3a, c, and measurements at the different temperatures *T*≈15 mK (a, d) and 60 mK (c, f) are displayed.”  is replaced by  “Measurements at the different temperatures *T*≈15 mK (a, d) and 60 mK (c, f) are displayed, as well as measurements at 35 mK obtained using the other (bottom) source QPC located on the opposite side of the analyzer (b, e) (see Supplementary Information for a different gate voltage tuning of the device that exhibits a behavior symmetric in the polarity of the bias for both source QPCs).”

In the Methods Section, subsection “Andreev observations for different temperatures and tunings”, the sentence “The main changes compared to Fig. 3c are that a different QPC (located on the opposite side of the analyzer) is used for the source, and the additional temperatures of *T*≈15 mK and 60 mK.” is replaced by “The main changes compared to Fig. 3c are the additional temperatures of *T*≈15 mK and 60 mK in Fig. 9a, d, c, f, and that a different QPC (located on the opposite side of the analyzer) is used for the source in Fig. 9b, e (see Supplementary Information for further data in the Andreev configuration).”

This has been corrected in both the PDF and HTML versions of the Article.

The “Data availability” statement in the original version of this Article did not contain a link to the data repository. This has now been corrected in both the PDF and HTML versions of the Article, which now include a link to the data repository containing data from the raw measurements, the data analysis code, and the plotted data. The new Data availability statement reads:

“The raw measurements used in this article, the codes to analyze these measurements, and the data plotted in the figures are available via *Zenodo* at 10.5281/zenodo.10091819.”

The original version of this Article did not include the Supplementary Information. The HTML version of the Article has been updated to include the Supplementary Information, which contains a figure showing data measured with a different gate voltage tuning of the device, more symmetric in bias voltage, to further support the findings in the main Article.

### Supplementary information

Supplementary Information

