# Supplementary material for: Quasiparticle Andreev scattering in the ν = 1/3 fractional quantum Hall regime
Source: Nat Commun. 2023 Jan 31;14:514. doi: 10.1038/s41467-023-36080-4 (PMC9889737; doi:10.1038/s41467-023-36080-4)
Supplement: Supplementary file 1 — Supplementary Information [file 41467_2023_36080_MOESM1_ESM.pdf]

Supplementary Information: Quasiparticle Andreev scattering in the $\nu = 1/3$ fractional quantum Hall regime

P. Glidic,[1] O. Maillet,[1] C. Piquard,[1] A. Aassime,[1] A. Cavanna,[1] Y. Jin,[1] U. Gennser,[1] A. Anthore,[1,2] and F. Pierre[1]

[1] *Université Paris-Saclay, CNRS, Centre de Nanosciences et de Nanotechnologies, 91120, Palaiseau, France*

[2] *Université Paris Cité, CNRS, Centre de Nanosciences et de Nanotechnologies, F-91120, Palaiseau, France*

In this supplementary information, we present additional observations of quasiparticle Andreev scattering obtained with a distinct gate voltage tuning of the device than in the main article, but at the same reference temperature $T \approx 35\,\mathrm{mK}$. Compared to the data in the main article, the Andreev signal obtained using the bottom source remains here more symmetric with the polarity of the bias, as seen from the comparison between Fig. 9e and Supplementary Fig. S1c. Consequently, the comparison testing the additivity between both sources active separately or together, which is observed at the same precision level, is also more symmetric (see Fig. 4 vs Supplementary Fig. S1f).

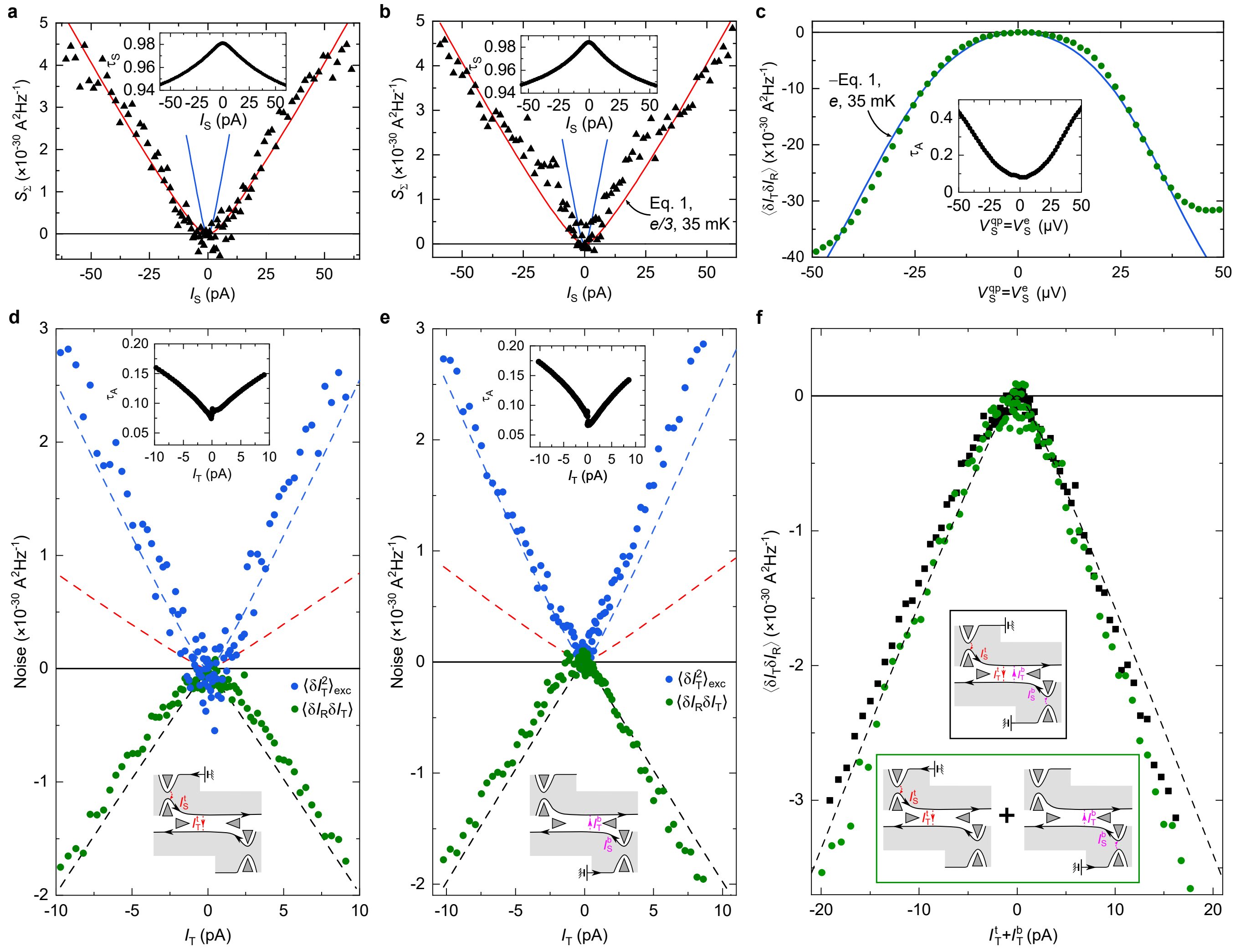

Figure S1. **Additional Andreev observations** with a distinct gate voltage tuning of the device. Symbols are data points at $T \approx 35\,\mathrm{mK}$. The continuous and dashed lines display the noise predicted by ±Eq. 1 and ±Eq. 5, respectively, for a tunneling charge e (blue), $e/3$ (red) and for the Andreev cross-correlations (black). Panels (d) and (e) display the noise signature of quasiparticle Andreev reflection using, respectively, the top and bottom QPC as the active quasiparticle source whose simultaneous characterization is shown in the corresponding above panel (a,b). The separate characterisation of the analyzer QPC (performed applying a direct voltage bias, see schematic in Fig. 2) is displayed in (c) . The Andreev additivity is observed in (f), where the data summing the two single source signal is shown green (sum of cross-correlations data in (d,e)) whereas the data measured with both sources active simultaneously is shown black.
